# Medication administration error reporting and associated factors among nurses working at the University of Gondar referral hospital, Northwest Ethiopia, 2015

**DOI:** 10.1186/s12912-016-0165-3

**Published:** 2016-07-18

**Authors:** Berhanu Boru Bifftu, Berihun Assefa Dachew, Bewket Tadesse Tiruneh, Debrework Tesgera Beshah

**Affiliations:** Department of Nursing, University of Gondar College of Medicine and Health Science, P. O. Box: 196, Gondar, Ethiopia; Department of Epidemiology and Biostatistics, Institute of Public Health, University of Gondar College of Medicine and Health Science, P. O. Box: 196, Gondar, Ethiopia

**Keywords:** Errors reporting, Medication administration, Nurses

## Abstract

**Background:**

Medication administration is the final step/phase of medication process in which its error directly affects the patient health. Due to the central role of nurses in medication administration, whether they are the source of an error, a contributor, or an observer they have the professional, legal and ethical responsibility to recognize and report. The aim of this study was to assess the prevalence of medication administration error reporting and associated factors among nurses working at The University of Gondar Referral Hospital, Northwest Ethiopia.

**Methods:**

Institution based quantitative cross - sectional study was conducted among 282 Nurses. Data were collected using semi-structured, self-administered questionnaire of the Medication Administration Errors Reporting (MAERs). Binary logistic regression with 95 % confidence interval was used to identify factors associated with medication administration errors reporting.

**Results:**

The estimated medication administration error reporting was found to be 29.1 %. The perceived rates of medication administration errors reporting for non-intravenous related medications were ranged from 16.8 to 28.6 % and for intravenous-related from 20.6 to 33.4 %. Education status (AOR =1.38, 95 % CI: 4.009, 11.128), disagreement over time - error definition (AOR = 0.44, 95 % CI: 0.468, 0.990), administrative reason (AOR = 0.35, 95 % CI: 0.168, 0.710) and fear (AOR = 0.39, 95 % CI: 0.257, 0.838) were factors statistically significant for the refusal of reporting medication administration errors at *p*-value <0.05.

**Conclusion:**

In this study, less than one third of the study participants reported medication administration errors. Educational status, disagreement over time - error definition, administrative reason and fear were factors statistically significant for the refusal of errors reporting at *p*-value <0.05. Therefore, the results of this study suggest strategies that enhance the cultures of error reporting such as providing a clear definition of reportable errors and strengthen the educational status of nurses by the health care organization.

## Background

Patient safety is the freedom from accidental or preventable injuries produced by medical care [1]. It is a central concern and an indicator of health care quality services [2]. That is why, concerns about patient safety have prompted the formation of a World Health Organization (WHO) patient safety program in response to a World Health Assembly in 2002 with the vision of ‘every patient receives safe health care, every time, everywhere [3].

Patient safety can be affected as a result of a constellation of different factors and circumstances [2]. In all health care systems, medical errors are the main factors endangering the patient safety [4]. Medication errors are one of the most common types of medical errors. Medication errors are an error in the medication process: ordering, transcription, dispensing, and administration [5]. Medication errors listed in the top ten causes of mortality worldwide [6]. A systematic review of 45 studies revealed that the prevalence of medication errors ranges from 2 to 75 % [7].

Medication administration error is one of the most common errors in the medication error process and occur when a discrepancy occurs between the drugs received by the patient and the drug intended by the prescriber [8]. For example, study from United Kingdom’s National Patient Safety Agency revealed that of different types of medication errors, almost 50 % account to administration error compared to 18 % for dispensing, and 16 % for prescribing [9]. A systematic literature review in Iran on medication error also indicated that reported prevalence of medication administration errors holds the highest ranges that is from 14.3 to 70 % compared to 29.8–47.8 % for prescribing error, 3–33.6 % for dispensing error and from 10 to 51.8 % for transcribing errors [10]. In Ethiopia, where there is lack of educated health care professionals and high patient flow, evidences indicate that medication error is a common problem that ranges from 4.35 to 89.9 % [11, 12]. For example according to a recently published study, [13] the incidence of medication administration error was 56.4 % of this, majority (87.5 %) indicated documentation error, followed by technique error 73.1 % and time error 193 (53.6 %) respectively. Ages, work experience, nurse to patient ratio, medication administration at night shift were associated with errors [13].

In health care delivery system, the outcome of any errors result in serious patient outcomes [14] in terms of morbidity, mortality, adverse drug events, re-admissions to hospital, and increased length of hospital stay [13, 15, 16]. Therefore, prompt detection and report of an error can reduce the risk of such serious patient outcomes [17]. Voluntary medication error reporting systems rely on the ability and willingness of individual physicians, pharmacists, and nurses to detect and report errors as part of routine practice [18]. Nurses are intimately involved in and ultimately responsible for the delivery of medication [19]. The medication administration process is a daily component of nursing practice and is often viewed as a routine and basic nursing task that account for around 40 % of their work time [15].

As part of the health care team, whether the nurses are the source of an error, a contributor, or an observer, they have a professional responsibility to recognize and report medication administration errors that could harm patient safety by clarifying ambiguous orders; and questioning orders that are inappropriate [14]. Error reporting through established systems provides opportunities to prevent future similar and perhaps even more serious errors, [9] nevertheless nurses are reluctant to report medication errors because of several reasons. Including: fear of disciplinary actions [20], lack of protection for reported errors, culture of blame and punishment, variations in how errors are defined and because of its potential damage to the hospitals’ reputation [4, 7, 9, 18, 20–22]. Regarding the association of medication administration error reporting and nurses characteristics, most of the finding indicated there is no relationship between medication administration error reporting and nurses’ characteristics. For example a survey of nurses revealed nurses did not vary in their concerns medication administration error reporting based on their socio demography such as age of the nurse, type of education and length of experiences [23, 24], married nurses and nurses on permanent contract [25]. Few study reveal inconsistent relation between medication administration error reporting and nurses’ characteristics such as: experience, education, sex, working position, working unit [26, 27]. Therefore, the main aim of this study was to assess medication administration error reported and associated factors among nurses working at the University of Gondar Hospital.

## Methods

### Study design, periods and study area

Institution based cross sectional quantitative study design was employed from May, 1 to 30, 2015 at The University of Gondar Hospital. The University of Gondar hospital is located at 748 km away from the capital city of Ethiopia, Addis Ababa. It is a tertiary level referral hospital, which acts as the referral centre for four district hospitals in the area and has 500 inpatient beds, and 559 health professionals to provide health service to the community of which the majority of them are nurses (*n* = 302). The hospital provides health referral services for over 5 million inhabitants in the North West region of Ethiopia.

### Participants

The participants of this study were all nurses working in The University of Gondar Hospital. The inclusion criteria were those nurses who directly involved in patient care and had been employed for more than six months. Head nurses and those in higher administrative positions were excluded.

### Instruments

For the assessment of medication administration errors reporting, we used medication administration errors reporting questionnaire. It contained 65 questions with three sections; the first section included 29 items regarding reasons why medication errors occur, second section included 16 items regarding reasons why medication errors not reported. Respondents were asked to indicate their level of agreement using a five point Likert type scale with fix values ranging from 5 = strongly agree to 1 = strongly disagree. The third section included 20 items regarding what percentage of each type of medication error actually reported in their units; 9 items for non intravenous (Non-IV) medication errors and 11 items for intravenous (IV) medication errors. More specifically, participants were asked to use a 10-point ordinal scale to indicate the range of MAEs which they perceived to be reported on their patient care units. This scale has been developed by Wakefield, et al., 2005. The items in the original instrument underwent rigorous validation and tested for its psychometric property factors analysis. The construct and criterion-related validity test and subscale reliability with Cronbach’s alpha were ranging from .69 to .76 [24, 28]. This scale has been widely used across the world including Africa [22, 24]. In this study, the reliability of the questionnaire was measured by Cronbach’s alpha and it had 0.81 for the 20 item MAER and 0.83 for the 16 item barrier to MAER.

For the purpose of this study, we used the estimated percentage of medication errors actually reported with 20 item and reasons why medication errors are not reported with16 items. The estimate of medication error reporting was defined by the estimated mean percentage of errors reported on the 20 items of medication errors that has actually been reported. This scale has been widely used across the world.

### Data collection methods

Data were collected using a semi-structured self administered questionnaire consists of socio-demographic characteristics and medication administration errors reporting questionnaire. The questionnaires were distributed in the nursing office by two MSc nurses working in the academic area. The questionnaires need 10–20 min to complete.

### Data processing and analysis

Data cleanup and cross-checking were made before the analysis. EPI info version 3.5.3 statistical software and Statistical Package for Social Science (SPSS) windows version 20 programs were used for data entry and analysis respectively. Bivariate and multivariate logistic regression and odds ratio with 95 % confidence interval were used to identify the associated factors with medication administration errors reporting. Variables with *p*-value (*p* < 0.05) were used as the cutoff point.

## Results

Total of 282 participants participated in this study with a 96.9 % response rate.

### Socio-demographic characteristics of the respondents

The majority of the participants were men 160 (56.7 %). The mean (± standard deviation) age of the participants was 28.89 (±9.70) years, 203 (72 %) of the participants were BSc in Nursing. Regarding their work experience, the majority of the participants 158 (56 %) served from 6 months to 4 years (Table 1).

**Table 1:** Socio-demographic characteristics of the respondents, Gondar University Referral Hospital (GURH), Northwest Ethiopia, 2015 (*n* = 282)

Characteristics	Number	Percent
Sex		
Male	160	56.7
Female	122	43.3
Age		
18–24	44	15.6
25–34	190	67.4
35–44	33	11.7
> = 45	15	5.3
Educational status		
Diploma	67	23.8
BSc in Nursing	203	72
MSC in Nursing	12	4.3
Religion		
Orthodox	242	85.8
Muslim	30	10.6
Protestant	7	2.5
Catholic	3	1.1
Marital status		
Single	159	56.4
Married	101	35.8
Divorced/Widowed	22	7.8
Work experience in years		
≤4	158	56
5–10	77	27.3
11–14	7	2.5
> = 15	40	14.2

### Perceived prevalence of medication administration error reporting

The estimated medication administration error reported was found to be 29.1 %. The perceived rates of medication administration error reported for non intravenous medications were ranged from 18.1 to 28.4 % and from 20.6 to 33.7 % for intravenous related medication (Table 2).

**Table 2:** Proportion of perceived medication administration error reported for each item at GURH, Northwest Ethiopia, 2015 (*n* = 282)

Type of Medication Error		Percentage of Each Type of Medication Error Actually Reported	
		Intra Venous (IV) Error	Non-Intra Venous (IV) Error
1	Wrong route	20.6	26.2
2	Wrong time	33.3	27.7
3	Wrong patient	20.6	18.8
4	Wrong Dose	30.5	19.1
5	Wrong drug	28.4	18.8
6	Medication is omitted.	30.1	28.4
7	Medication is given, but not ordered by physician.	20.9	24.8
8	Medication administered after the order discontinued.	33.7	23.4
9	Given to patients with a known allergy.	22.7	18.1
10	Wrong fluid	22.3	----
11	Wrong rate	23.8	----

### Reasons for Not reporting medication administration errors

The overall mean and standard deviations of the subtotal scores were: 19 ± 8.14 for disagreement over time and error definition reasons, 12.03 ± 5.37 for fear reasons and 11.93 ± 5.19 for administrative reasons.

### Factors associated with medication administration error reporting

From the bivariate analysis: sex, age, educational statuses, work experience, disagreement over time and error definition, administrative and fear reason were factors associated with the refusal of MAEs at *p*-value <0.2 and entered into multivariate analysis. From the multivariate analysis; Education status (AOR =1.38, 95 % CI: 4.009, 11.128), disagreement over time - error definition (AOR = 0.44, 95 % CI: 0.468, 0.990), administrative reason (AOR = 0.35, 95 % CI: 0.168, 0.710) and fear (AOR = 0.39, 95 % CI: 0.257, 0.838) were factors statistically significant for the refusal of reporting medication administration errors at *p*-value <0.05 (Table 3).

**Table 3:** Bivariate and multivariate logistic regression analysis of factors associated with MAER at GURH, Northwest Ethiopia, 2015 (*n* = 282)

Explanatory variables	MAER		COR (95 % CI)	AOR (95 % CI)	*P*-value
	Yes N (%)	No N (%)			
Sex					
Male	38 (23.8)	122 (76.2)	0.55 (1.078,3.043)		
Female	44 (36.1)	78 (63.9)	1		
Age					
20–24	10 (22.7)	34 (77.3)	1		
25–34	52 (27.4)	138 (72.6)	1.28 (0.360,1.1.692)		
35–44	15 (45.5)	18 (54.5)	2.83 (1.132,5.943)		
>/=45	5 (33.3)	10 (66.7)	1.7 (0.163,2.125)		
Educational status					
Diploma	11 (16.4)	56 (83.6)	1	1	
BSc and above	71 (33)	144 (67)	2.51 (2.188,9.775)	1.38 (4.009,11.128)	<0.001
Work experience in year					
</=4	39 (24.7)	119 (75.3)	1		
5–10	23 (29.9)	54 (70.1)	1.30 (0.419,1.412)		
11–15	5 (71.4)	2 (28.6)	7.63 (0.024,0.703)		
>/=16	15 (37.5)	25 (62.5)	1.83 (0.262,1.139)		
Disagreement over time - error definition					
Agree	38 (33.1)	89 (66.9)	1	1	1
Disagree	44 (25)	111 (75)	0.93 (0.201,0.778)	0.44 (0.468, 0.990)	<0.001
Administrative reason					
Agree	47 (33.1)	95 (66.9)	1	1	1
Disagree	35 (25)	105 (75)	0.67 (0.401,0.978)	0.35 (0.168, 0.710)	0.004
Fear reason					
Agree	40 (26)	87 (74)	1	1	1
Disagree	43 (31.6)	112 (68.4)	0.84 (0.451,0.767)	0.39 (0.257, 0.838)	0.006

## Discussion

The main purpose of this study was to assess medication administration error reporting and associated factors. Finding from this study revealed that the estimated MAER was found to be 29.1 %. This finding support the study carried out in Korea and Taiwan that revealed the prevalence of 28.3 % [29] and 24.5 % [30] respectively. But the result of this study (29.1 %) is slightly higher than the study carried out in Saudi Arabia 22 % [24]. On the other hand compared to other study carried out in Jordan, 86 % [31] and 35 % [32] and 48 to 70 % in Taiwan [23], the result of this finding (29.1 %) is lower. The possible reason for the difference may be accounted for fear of legal issues, blame for the reported errors in the working environment and lack of personal confidence to withstand any punishments following the reporting of errors. Study from United State revealed that the main reasons for nurses do not report medication administration errors were: fear, disagreement over whether or not an error had occurred, administrative responses to medication errors, and the effort involved in the reporting process [33]. Another study also revealed that around 25 % of the study participants did not reported error because of fear of their supervisors punish [34].

Regarding factor or barrier for MAER using MAER questionnaires, disagreement over time - error definition is considered as the most perceived barrier followed by fear reasons and administrative reasons for MAER. This finding is supported by several other studies using similar instrument as disagreement over time – error definition, fear reasons and administrative reasons are barrier for MAER with different ranking order [22, 23, 30, 31]. As to the statistical association, those participants who disagreed for the presence of disagreement over time - error definition as a barrier for MAER were about forty four times more likely reported MAE (AOR = 0.44, 95 % CI:0.468, 0.990) than those participants who agreed for the presence of disagreement over time - error definition as a barrier for MAER. This finding is similar to other studies [35]. This could be due to the fact that those participants who do not clearly recognized the actual definition of medication administration error do not report the error because reportable errors need their own knowledge like the knowledge of error, who it could be reported, to whom it reported and the like. Studies support this explanation as a knowledge deficit is seen in lack of knowledge of process (not knowing how to report an error) and uncertainty about definitions (e.g., what is an error? What is a near miss?) for the refusal to report errors [22, 36]. Another cross-sectional study from Korea revealed that although 95 % of participants were not afraid to report mistakes, but about half of the respondents reported that they were not clear about the types of errors should be reported [37].

Those participants who disagreed administrative reason as a barrier for MAER were thirty five times more likely reported MAE (AOR = 0.35, 95 % CI: 0.168, 0.710) than those participants who agreed administrative reason as barrier for MAER. In this study more than half (50 %) of the study participant did not reported MAE because of Administrative Reason. This is similar with other studies [22, 23, 37].

Those participants who disagreed fear reason as a barrier for MAER were about thirty nine times (AOR = 0.39, 95 % CI: 0.257, 0.838) more likely reported MAE than those participants who agreed fear reason as barrier for MAER. This may be due to the fact that fear of the consequence of the errors on their future career. This result is consistent with other studies [16, 22, 23, 30, 31].

Those participants who had educational status of BSc and above were more than one times (AOR =1.38, 95 % CI: 4.009, 11.128) more likely reported MAE than those participants who had educational status of diploma. These result is consistent with the previous study. This is due to the fact that those participants who had higher educational status may have higher knowledge, attitude and practice toward the drug adverse effect or they may develop confidence to defend the consequence of MAER through their educational journey.

### Limitation of the study

This study has some important limitations that should be kept in mind when interpreting the results. The cross-sectional nature of the study design does not confirm definitive cause and effect relationship and since, the study was based on self-reported information that may be prone for reporting bias because of the respondent’s interpretation of the questionnaire or desire to report their feeling. Some nurses characteristics such as working area (unit), working position and other were not collected that may affect MAE reporting.

## Conclusion

Overall, more than two third of the participants did not reported medication administration errors. Educational status, disagreement over time - error definition, administrative and fear reason were factors statistically significant for the refusal of reporting medication administration errors at *p*-value <0.05. Therefore, the results of this study suggest strategies that enhance the cultures of error reporting such as providing a clear definition of reportable errors, establishing a good relationship with the healthcare administrators that make the workers free to report any mistakes without fear and strengthen the educational status of nurses by the health care organization.

## Abbreviations

AOR, adjusted odd ratio; CI, confidence interval; COR, crude odd ratio; MAER, Medication Administration Error Reporting; Non-IV, non intravenous; SPSS, statistical package for social science; WHO, World Health Organization
